# Reduction in hSOD1 copy number significantly impacts ALS phenotype presentation in G37R (line 29) mice: implications for the assessment of putative therapeutic agents

**DOI:** 10.1186/1477-5751-13-14

**Published:** 2014-08-08

**Authors:** Pierre Zwiegers, Grace Lee, Christopher A Shaw

**Affiliations:** 1 Program in Experimental Medicine, University of British Columbia, Vancouver, Canada; 2 Division of Neurology, Department of Medicine, University of British Columbia, Vancouver, Canada; 3 Department of Ophthalmology and Visual Sciences, University of British Columbia, Vancouver, Canada; 4 Program in Neuroscience, University of British Columbia, Vancouver, Canada

**Keywords:** SOD1, ALS, Copy number variation, G93A, G37R

## Abstract

**Background:**

*In vivo* animal models of familial amyotrophic lateral sclerosis (fALS) are widely used to delineate the potential role that genetic mutations play in the neurodegenerative process. While these models are extensively used for establishing the safety and efficacy of putative therapeutics during pre-clinical development, effective clinical translation of pharmacological interventions has been largely unsuccessful.

**Results:**

In this report we compare a recent cohort of G37R (line 29) mice generated from mating wild-type females with transgenic males obtained commercially to a previous set of offspring produced with transgenic male breeders from a colony established at a local collaborator’s facility. Commercially derived progeny presented with a tightly clustered genomic signature for the mutant human superoxide dismutase1 transgene (hSOD1) locus, and exhibited a greater than two-fold reduction in the number of transgene copies present in the genome compared to offspring derived locally. Decrease in transgene levels corresponded with delayed ALS progression and a significant increase in overall lifespan (146%).

**Conclusions:**

These results highlight some key challenges inherent to the use of G37R (line 29) animals in pre-clinical studies for the development of ALS therapeutics. Without stringent assessment of mutant SOD1 copy number/protein levels, heterogeneity of transgene levels within cohorts may influence the behavioural and pathological presentation of disease and thus calls to question the validity of any detected therapeutic effects. Nuanced changes in mutant SOD1 copy number that currently remain unreported may undermine research endeavours, delay efforts for clinical translation, and compromise the rigor of animal studies by limiting reproducibility amongst research groups.

## Background

Amyotrophic Lateral Sclerosis (ALS) is a progressive paralytic disorder of unknown etiology marked by the degeneration of motor neurons in the brain and spinal cord. Approximately 5% of reported familial ALS cases (fALS) are linked to genetic mutations and are inherited in an autosomal dominant fashion [1]. Less than 20% of these are linked to over 170 toxic gain-of-function mutations in any of the five exons of the gene encoding the Cu/Zn superoxide dismutase 1 (SOD1) enzyme [2], albeit it a loss-of-function may also contribute to ALS pathobiology [3]. Although the vast majority (90%) of ALS is sporadic (sALS) and has no known cause, these cases have been reported to be clinically and neuropathologically indistinguishable from the inherited forms of the disease [4].

Animal models delineating disease-course specific parameters provide a glimpse into the mechanism(s) of disease pathogenesis and can serve as a platform to explore etiological agents that augment disease presentation. Transgenic mouse models developed to harbour mutations found in fALS patients mimic the progressive nature of the disease, aspects of motor neuron death, and histopathological hallmarks associated with clinical ALS [5,6].These animal models have been crucial in demonstrating that it is not a reduction of dismutase activity *per se* that predisposes the system to an ALS phenotype, but rather the acquisition of novel toxic properties in the SOD1 protein product [7]. A recent study using the G37R mouse model demonstrated that the fALS phenotype and related histopathology is enhanced with an environmental agent that is sufficient at producing a neurodegenerative phenotype alone [8]. The ability of *in vivo* models to provide a glimpse into the mechanistic underpinnings of disease pathogenesis underscores their value in the study of neurodegenerative disorders and ultimately in translational research for therapeutic development.

Several transgenic mouse models of fALS have been generated which include animals harbouring the G93A [9], G37R [10], G85R [11], and D90A mutations [12]. While all of these mouse models are characterized by motor neuron loss and similar histopathological findings, the G93A and G37R mutations are currently the most widely used and have produced the most detailed phenotypic data (Tables 1 and 2).

**Table 1:** Progressive phenotypic correlates in the G93A murine model of ALS1

					**Age at pathology (d)**		
**Genetic modification**	**# of gene copies**	**Relative SOD1 Protein Levels (brain) (ng SOD1 protein/μg tot soluble brain protein)**	**Relative increase in SOD1 activity (U/μg soluble brain protein)**	**Mean ALS onset (d)**	**Mean age at death (d)**	**Motor neuron vacuolation**	**Motor neuron loss**
Genomic hSOD1 G93A (G1) [5,9,13,14]	18	2.2	2.56	90-120	153-185	73-163	>180
Genomic hSOD1 G93A (G1H) [5,7,15]	25	3.36	3.04	90	136	37	80
Genomic hSOD1 G93A (G1L) [16]	13	n/a	n/a	200	251	200*	230
Genomic hSOD1 G93A (G5/G5) [7]	10	1.28	1.61	300	>400	**	>300

*V. Little evidence of vacuolar pathology at this late stage; **V. Little vacuolar pathology evident at end stage of phenotype.

**Table 2:** Progressive phenotypic correlates in the G37R murine model of ALS1

				**Age at pathology (d)**		
**Genetic modification**	**Relative SOD1 protein levels (human/mouse) spinal cord**	**Total increase in SOD1 activity**	**ALS onset (d)**	**Motor neuron vacuolation**	**Motor neuron loss**	**Reactive astrogliosis**
Genomic hSOD1 G37R(42) [10]	12.3	14.5	106-121	77	105	77
Genomic hSOD1 G37R(9) [10]	6.2	9	152-183	70	105	35
Genomic hSOD1 G37R(106) [10]	5.3	7.2	167-228	189	126	77
Genomic hSOD1 G37R(29) [10]	5	7	183-243	none	133	77
Genomic hSOD1 wild-type (76) [10]	10.5	9.2	None by 548d	none	none	none

Originally developed by Wong *et al*. [10], the transgenic G37R line retaining full SOD1 specific activity was generated via PCR-directed mutagenesis. After microinjection into a fertilized murine embryo, multiple copies were randomly inserted into the genome; giving rise to several founder lines exhibiting varying degrees of mutant hSOD1 expression levels and the associated neuropathology (Table 2). Early studies of these lines showed a correlation between reduced expression levels of the mutant hSOD1 locus and an attenuated presentation of ALS-associated pathologies within a defined time frame [10].

Extended observations of additional founder lines which express markedly reduced levels of the mutant transgene fail to recapitulate any features of motor neuron degeneration and do not present with pathological outcomes [10]. This initial work correlating disease presentation with the degree of mutant hSOD1 expression has demonstrated that the aberrant phenotype is highly dependent upon the number of defective SOD1 copies randomly inserted into the genome when the transgenic line is established. In addition, mutant hSOD1 expression levels are affected by rare and spontaneous intralocus recombination events at the meiotic level which have been shown to reduce transgene copy number and result in the attenuated presentation of ALS-associated pathologies [7,17].

Although transgenic mice have been instrumental in delineating the underlying causal mechanism(s) of ALS pathogenesis, scientific data from research using mutant SOD1 mouse models may have limited translational applicability. A multitude of promising therapeutic strategies with demonstrated efficacy in the mouse have failed when assessed in human clinical trials [18,19]. Although transgenic mouse models recapitulate many prominent features of ALS, unlike clinical cases, each of these requires a significant overexpression of the mutant locus to elicit the neurodegenerative disease process, and is subsequently sensitive to transgene dosage effects [17]. Furthermore, given that such a small fraction of all ALS cases arise due to genomic anomalies in the hSOD1 locus, the best characterized mutant SOD1 models may have limited reliability for translational research on potential ALS therapies and questionable applicability to sporadic ALS research [19].

**Figure 1:**
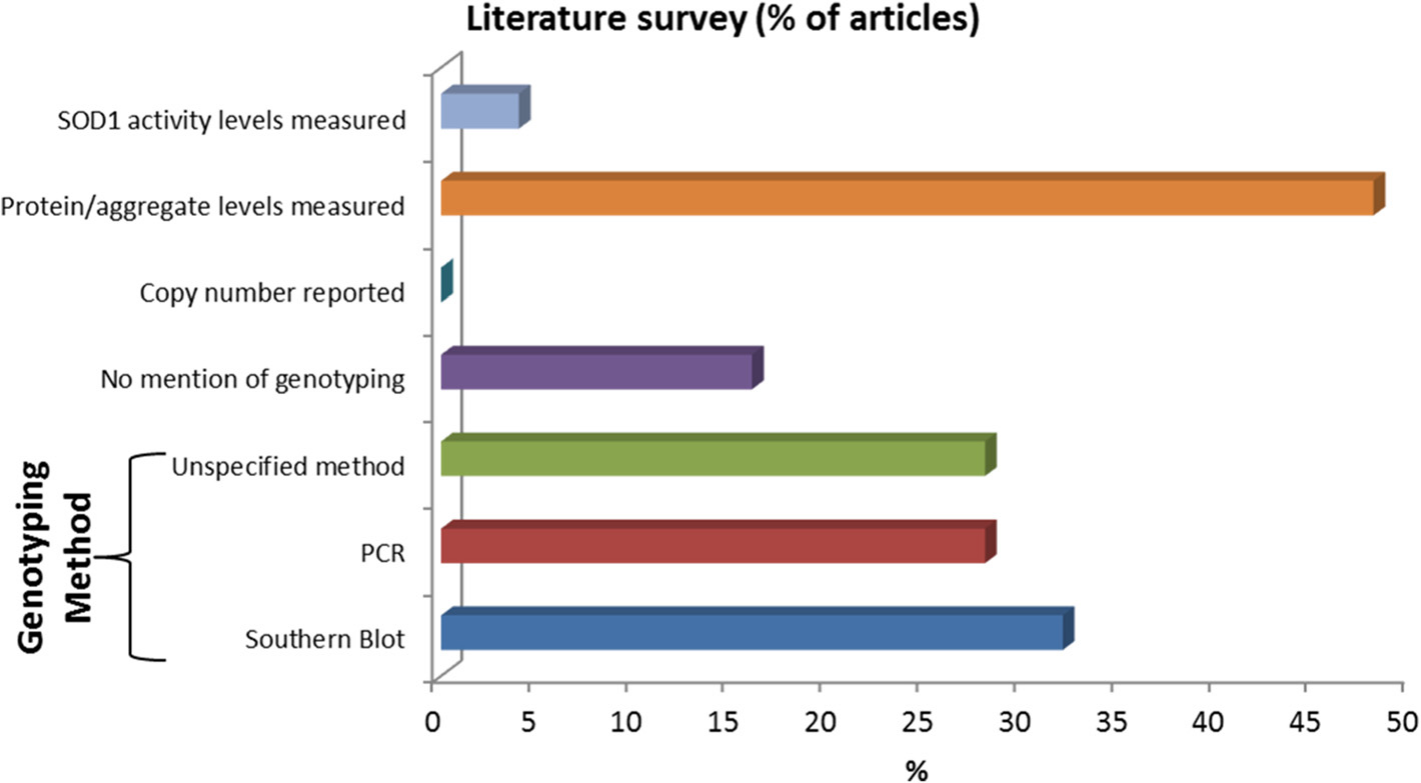
**Literature survey for details regarding the genomic SOD1 G37R locus.** A review of publications (n = 25) listed on the Mouse Genome Informatics database over the preceding two decades in reference to G37R line 29 animals reveals: (1) discrete copy numbers have not been characterized, (2) SOD1 protein and/or aggregate levels are only assessed in half of the studies, and (3) given a genotype assessment, no values regarding relative transgene presence are reported.

To date, discrepancies between various pre-clinical efficacy studies using transgenic hSOD1 mice have hampered clinical translation of potential therapeutic compounds. [18-20]. A survey of 25 studies involving line 29 G37R animals over the past two decades indicates that crucial information such as transgene copy number or level of enzyme activity are not usually quantifiably determined or reported (Figure 1). Candid disclosure of these parameters is crucial in assuring the scientific rigor and replicability of results between various research groups and treatment modalities. In the absence of stringent experimental design and reporting parameters, the utility of these models in delineating potential therapeutic avenues may be limited [21,22].

To underscore these challenges, we report on the ALS phenotype of progeny derived from commercially obtained G37R (line 29) breeders with unexpected deficits in genomic mutant hSOD1 levels and discuss the potential implications of this data for the discovery of effective ALS therapies. We intend that the results described in this report will stir discussion on the appropriate considerations for ALS research using transgenic hSOD1 mice in an evolving clinical therapeutics context.

## Results

### Varied hSOD1 transgene levels in collaborator- and commercially derived G37R (line29) progeny

**Figure 2:**
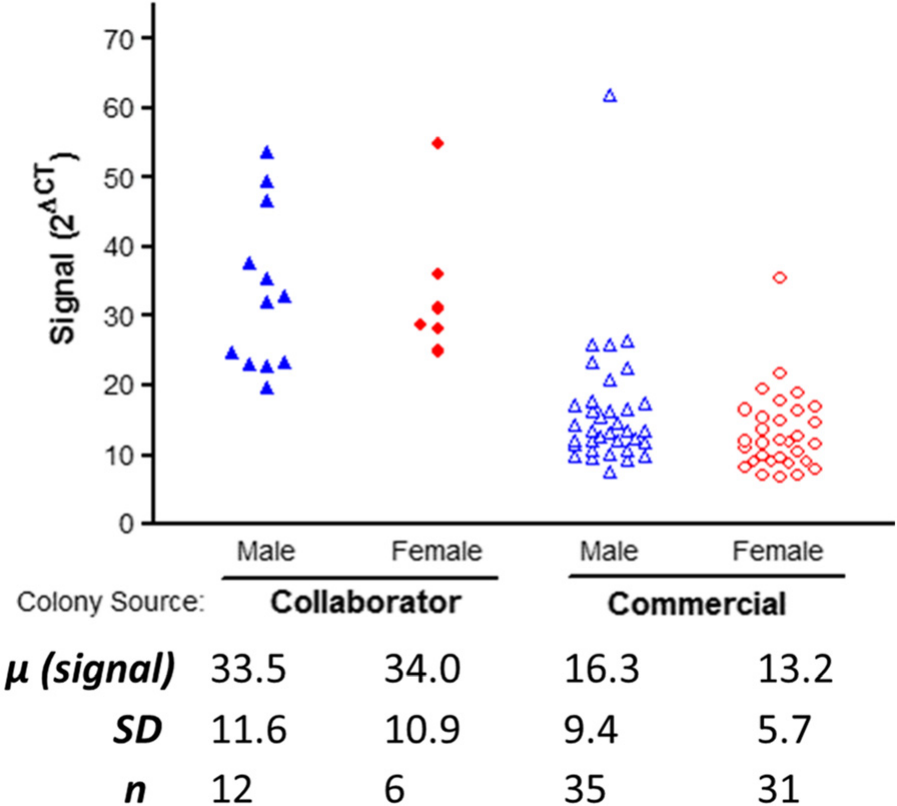
**Comparisons of qualitative genotyping for the mutant hSOD1 locus in two independently-derived colonies.** Progeny derived from commercial breeders exhibited more than a two-fold decrease in the genomic G37R signature compared to collaborator derived offspring. Results are presented as the relative difference between the PCR cycle threshold (CT) values of the housekeeping (CJUN) and target (mutant SOD1) genes, raised to the second power. Kruskal-Wallis non-parametric analysis followed by Dunn’s Multiple Comparison test, indicated significant inter-source differences (p < 0.001, 0.01) while failing to differentiate loci heterogeneity from within each source. Collaborator- ♂ vs. Commercial-♂ (p < 0.001); Collaborator- ♂ vs. Commercial-♀ (p < 0.001); Collaborator- ♀ vs. Commercial-♂ (p < 0.01); Collaborator- ♀ vs. Commercial-♀ (p < 0.001).

Progeny generated from breeders obtained from a local collaborator demonstrated a mean signal (2^ΔCt^) of 33.5 (males) and 34.0 (females), while the signal from offspring generated from commercial breeders was 16.3 (males) and 13.2 (females); with a distribution that was relatively tightly clustered (Figure 2). Thus, the cohort of line 29 progeny that our group had generated from commercially obtained G37R male breeders exhibited more than a two-fold decrease in the relative hSOD1 copy number for both sexes when compared with progeny derived from a collaborator’s breeding colony (Figure 2). Differences in the relative abundance of the transgene locus between sexes within either progeny group were not statistically significant (Figure 2). Conversely, both intra- and inter-sex comparisons between progeny groups revealed a significant relative difference in transgene copy number, with commercially derived progeny exhibiting a significantly diminished genomic hSOD1 signature (Figure 2; p < 0.01 or p < 0.001).

### Delayed symptom onset and increased lifespan correspond to reduced transgene levels

**Figure 3:**
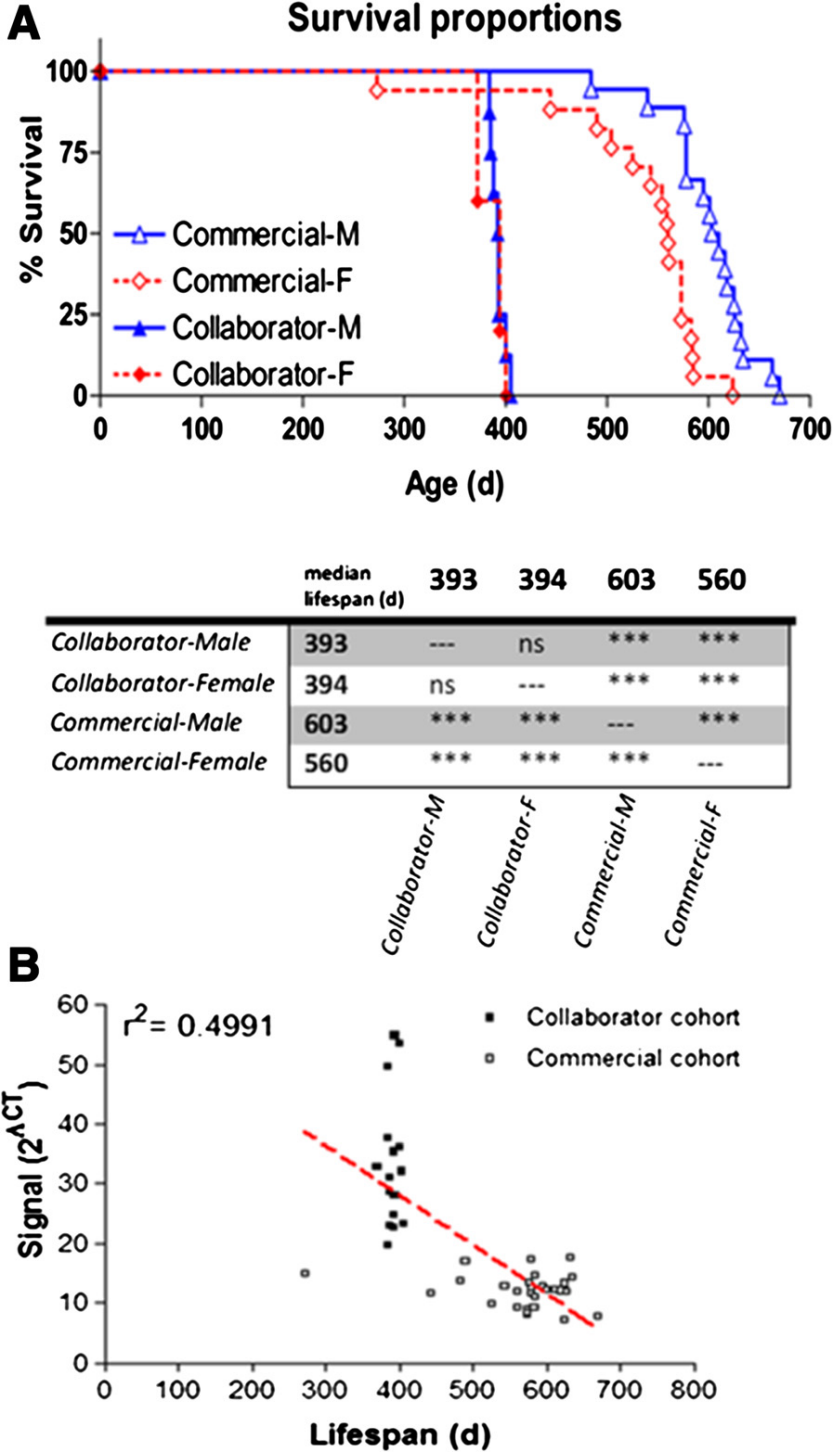
**Transgene dosage effects on life span. (A)** Kaplan-Meier survival analysis. Transgenic animals with a lower CT signal value exhibited an increased survival time. Bonferroni correction for multiple comparisons indicated a significant difference between animal source and lifespan. Inter-sex comparisons show that collaborator derived progeny with a higher CT signal value reached the endpoint (inability to self-correct) after one year of age, while commercially derived progeny with a lower CT signal value reached the endpoint much later, beyond 1.5 years of age. ***p < 0.001. Collaborator- ♂ n = 8; Collaborator- ♀ n = 5; Commercial-♂ n = 18; Commercial-♀ n = 17. **(B)** Linear regression analysis reveals a negative correlation between relative mutant SOD1 copy number and lifespan (p < 0.0001). Collaborator n = 16; Commercial n = 28.

**Figure 4:**
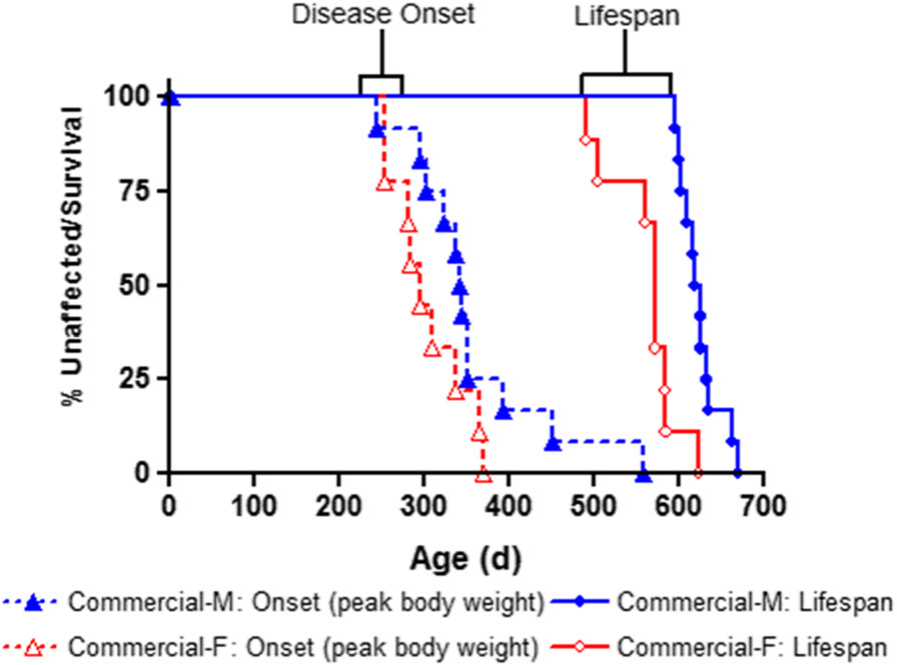
**Disease progression in commercially derived transgenic progeny.** Kaplan-Meier analysis did not indicate a significant difference between sexes for disease onset (p = 0.0926), although presentation of disease symptoms was slightly later in males. Males exhibited an increased lifespan compared to females (p < 0.0001). Commercial Male n = 12; mean age of onset/lifespan: 357/624d; Commercial Female n = 9 mean age of onset/lifespan: 304/563d).

Due to the temporal progression of disease phenotype in affected mice, neuronal degeneration ultimately results in severe paralysis of the hind limbs and progresses to a humane experimental endpoint at which the animal can no longer self-correct after being placed on its side. Comparing the survival curves between collaborator- and commercially derived progeny indicates that the relative amount of hSOD1 transgene copies within the genome can have a significant effect on the presentation of an ALS phenotype. Kaplan-Meier survival analysis indicated a 146% increase of mean lifespan in commercially derived progeny compared to offspring generated from a collaborator’s colony (Figure 3A). Linear regression analysis demonstrates a slightly negative correlation between the abundance of hSOD1 transgene and overall subject longevity (r^2^ = 0.4991, significant non-zero slope: p < 0.0001) (Figure 3B).We defined disease onset as the time point at which each animal had reached its peak body mass prior to progressive disease-related atrophy and loss of body mass. Although commercially derived male progeny exhibited a significant increase in lifespan compared to female mice, there was no difference in disease onset between sexes (Figure 4). Additionally, males and females derived from commercial breeders exhibited a similar delay between ALS onset and termination (Figure 4; 267 days for males, 259 days for females).

## Discussion

We identified several deviations from the expected onset and progression of disease in mutant SOD1 G37R (line 29) progeny derived from the mating of commercial transgenic male breeders with congenic C57BL/6 females, and explored sex differences within these deviations.

All transgenic G37R progeny generated from commercial male breeders exhibited more than a two-fold reduction in transgene dosage, a significant delay in ALS onset, and an attenuated rate of disease progression compared to offspring derived from a colony of male breeders obtained locally (Figures 3 and 4). Male and female progeny from the commercially derived group exhibited ALS-like outcomes at a similar time point. However, females experienced a steeper rate of decline, and ultimately reached the humane end point for euthanasia at an earlier time. This observation is in contrast to other published works which indicate that female animals generally survive longer than their male counterparts [17,23].

We observed marked differences in the onset and progression of the diseased phenotype in our commercially generated progeny compared to those observed in other studies of G37R (line 29) animals. Under similarly defined parameters, Ezzi *et al.*[24] reported disease onset at 307 +/-6 days and a median lifespan of 376 days (2010) which is a markedly earlier age of onset and endpoint compared to our commercially derived cohort (onset at 335 +/-72 days; lifespan of 603 days; male and female groups combined).

Transgenic hSOD1 animals exhibit an ALS phenotype whose severity and timing depends on the number of tandem transgene copies that incorporate into the genome following microinjection into a fertilized egg [7,17]. Two explanations can account for the attenuated ALS phenotype observed in our commercially derived progeny. First, widespread transgene loss within the genome may have been mediated by rare intralocus cross-over events during meiotic recombination when the colony was established in our facility. In the G93A model, this is a relatively rare occurrence, and changes in the number of transgene copies can occur in approximately 3% of progeny generated [17]. Thus, 97 percent of all meiotic events resulting in transgenic G93A -and presumably G37R- progeny do not exhibit genomic loss of hSOD1 copies, and subsequently present with ALS symptoms within a well-defined time span. Additionally, employing multiple breeding pairs theoretically diminishes the possibility of this event occurring and should assure transgene level homogeneity throughout the colony.

Second, an alternate and perhaps more plausible explanation is that the original commercial male breeders used to generate the progeny may have been bred from a colony with a reduced hSOD1 copy number. This reduced genomic load of the hSOD1 transgene would then have been propagated though several rounds of breeding in our study (Jackson Laboratory, personal communication). However, this is enigmatic as all the original JAX^®^ breeders used in our study exhibited tightly clustered CT values that were similar to historical control samples from G37R (line 29) mice, indicating comparable transgene levels within the genome (Additional file 1: Figure S1). To our knowledge, no data have been published demonstrating the significantly attenuated ALS disease progression in an entire cohort of G37R (line 29) progeny bred from multiple breeding sets. This may be because this is a truly novel presentation that has never previously been observed through multiple rounds of breeding in other facilities. Alternatively, a lack of reported phenotypic deviations in the scientific literature may resemble a publication bias against data that do not conform to established parameters.

### Genomic variation in the G93A model

The G93A transgenic model of fALS is the most widely chosen system for evaluating the pre-clinical efficacy of potential ALS therapeutic agents owing to its rapid and robust phenotype [18].

Characterization of the various G93A murine models have clearly established that the number of transgene copies randomly inserted into the genome can exert a pronounced effect on the onset and rate of disease progression. Line G1, with 18 G93A hSOD1 genomic copies, exhibited the greatest increase in SOD1 enzymatic activity, showed evidence of motor deterioration by approximately 3 months of age, and reached an end state of hind limb paralysis at 150 days of age [9]. On the other hand, line G20, with approximately 2 hSOD1 genomic copies exhibited a 6-fold decrease in total SOD1 protein levels compared to the G1 line and expressed a markedly delayed onset in disease phenotype [9].

Due to rare recombination events, the original G1 line has since been mated to produce two sub-lines that have either increased or decreased the number of hSOD1 copies randomly inserted into the genome [5]. A mouse line with a 40% increase in copy number (25 copies of the G93A locus) produced a more severe ALS pathology (Table 1; [15]), while a 30% reduction in copy number (13 copies of the G93A locus) resulted in an attenuated ALS phenotype as compared to the original G1 line (Table 1; [7]). These findings have further established the effect of transgene dosage and highlight the need for a deeper discourse on genetic information in studies that use transgenic animals to model ALS.

Variations in hSOD1 transgene within the G93A model have paved the way for creating alternate controlled systems to study the progression of ALS pathology. However, without crucial information regarding the abundance of hSOD1 sequences within the genome of transgenic ALS models and/or the expression levels of the locus, comparison of therapeutic efficacy studies between various research groups will continue to face discrepancies [21].

### Modeling ALS to test putative therapeutics

Critical assessment of this experimental paradigm in the study of ALS is the paramount focus of this discussion. Mutations in the SOD1 locus account for only a fraction of the population that are afflicted with ALS [1]. Nonetheless, researchers have largely focused on understanding SOD1-mediated mechanisms of disease and postulated that fALS and sALS arise from a common disease pathway [4]. Similarly, transgenic mutant hSOD1 animal models have gained widespread scientific interest for identifying and testing putative therapeutic agents for clinical ALS. Failure to demonstrate an efficacious treatment for ALS to date has cast doubt on the utility of preclinical data for therapeutic research [18]. In the discussion to follow, we present three possible explanations for this failure in therapeutic discovery.

#### Marked overexpression of mutant loci produces a severe phenotype that is unresponsive to treatment and may not be a reliable model for clinical ALS

As discussed previously, the onset and rate of disease progression in murine models are dependent upon transgene dosage. Thus, to produce a rapid and robust ALS phenotype, the number of transgene copies introduced into the genome need to be in excess of the endogenous locus, leading to a marked overexpression of mutant SOD1 products. Within the context of a significant overproduction of mutant SOD1 protein products, it may be unreasonable to suppose that any therapeutic agent can significantly impede the onset or progression of this experimentally induced form of ALS. Alternatively, it is possible that some pharmacological interventions which failed to progress past the pre-clinical stage may have been worth pursuing in further efficacy studies since fALS patients do not express the mutant SOD1 locus to the degree observed in transgenic mice with multiple hSOD1 genomic copies. Without significant pre-clinical evidence for treatment efficacy in these models, it is unlikely that therapeutic research will progress towards clinical applications.

Furthermore, potential therapeutics discovered in pre-clinical research may not adequately address the pathobiological mechanisms inherent to clinical ALS. For example, one of the main characteristics reported in animals with higher mutant hSOD1 levels is the marked vacuolar degeneration present in the spinal cord [5,10]. This type of neuronal pathology is not corroborated in post mortem fALS or sALS patient samples, and is typically explained as a toxic artefact of mutant SOD1 overexpression since transgenic animals with a lower copy number do not show vacuolar degeneration [5,7,14,25]. It is possible that vacuolar pathology in mutant hSOD1mice arises from overexpression of the transgene to produce a more severe disease pathology that is uncharacteristic of human ALS cases. When taken together, a more severe phenotype expected from greater quantities of mutant loci may not be a reliable model system to measure success or failure in translational therapeutic studies.

#### Genomic “noise” within animal cohorts may influence the course of disease progression and biases outcomes from therapeutics studies

Once the line is established, changes in genomic quantities of the mutant SOD1 transgene are relatively infrequent due to the improbability of intralocus meiotic recombination events. Experimental subjects with varying numbers of transgene copies will diverge along a spectrum of transgene expression. Outliers at either end of this spectrum will affect overall measures of severity of the observed ALS phenotype and may bias reports on the outcomes of pre-clinical therapeutic trials. For example, the presence of outliers with greater than average number of transgene copies tends to increase the estimate of ALS severity, thus decreasing the calculated efficacy of pre-clinical therapeutic tests and lowering the chance of discovering a potential treatment. Conversely, outliers occupying the opposite end of this spectrum would ultimately slant therapeutic tests towards reporting significant results, a false positive bias that has ethical, economic, and social consequences. If there is no diligent assessment of transgene dosage and/or mutant protein expression levels, it is likely that any therapeutic results observed may be primarily a function of mutant locus heterogeneity and the corresponding disease phenotype (Additional file 2: Figure S2). Hence nuanced changes in transgene levels that dictate the onset and progression of neurodegeneration may invalidate comparisons of experimental results between various research groups.

### fALS vs sALS

Mutations within the SOD1 locus account for nearly 1 in 5 of all fALS cases which translates roughly to 1% of all clinical presentations of ALS [1,26]. Researchers have proposed that fALS and sALS are clinically indistinguishable, and hence understanding the familial form will illuminate possible neuropathological mechanisms in sALS [27,28]. However, this view of an indistinguishable clinical overlap amongst cases of diverse etiologies is problematic as disease pathways leading to degenerative cascades may be different. For example, degenerating motor neurons in sALS lack evidence of epitope-specific labelling for misfolded SOD1 aggregates, and this suggest a pathological dissimilarity from familial forms of the disease [29]. Additionally, the discovery of pathological ubiquitinated TDP-43 inclusions, which are unique to sALS, further supports the view for divergent disease mechanism(s) for sALS and SOD1-related fALS [30]. Thus, pre-clinical studies of therapeutic efficacy in models of genetic causality may have implications for other genetic models of ALS, but are likely unable to provide meaningful results that are translatable to sALS.

## Conclusion

These results highlight key challenges for scientific rigor in using mutant hSOD1 G37R (line 29) genetic animal models to study putative ALS therapeutics and urge for the critical evaluation of using genetic animal models to test the efficacy of potential ALS therapies. More stringent criteria are needed to ensure homogeneity of transgene load and consistent SOD1 expression levels across research studies to allow reliable comparisons. This would identify sources of noise that, if included in data analysis, would introduce biased findings based on errors of standardization. Additionally, candid dissemination of unexpected findings to the broader research community is crucial to expediently redirect resources away from non-productive research endeavours where outcomes measuring therapeutic value are questionably biased by variability in transgene copy number. Of key interest is the tension between reports that fALS and sALS are clinically and pathologically similar, and evidence that they may arise from different disease pathways. Research priorities should now be established to develop appropriate models of sporadic disease to improve scientific understanding and refine therapeutic research efforts.

## Materials and methods

### Animal husbandry

All research methods involving animal research subjects were in accordance with the established guidelines of the Canadian Council on Animal Care, and by the Animal Care Committee of the University of British Columbia. All procedures involving mice were done at the Jack Bell Research Centre (Vancouver, British Columbia), in a controlled environment: 12-hour light/dark cycle and a temperature maintained at 21-22°C. Food (Purina LabDiet^®^) and water were provided *ad libitum*. Palliative care was initiated at the onset of minor gait perturbations, and mice were given a high caloric gelatinous substrate for nutrition and hydration.

All progeny were weaned at three weeks of age, and ear punch samples were collected to identify transgenic offspring via an automated genotyping platform (Transnetyx, TN). Samples were quantitatively genotyped against the c-Jun housekeeping gene for mutant SOD1 presence (forward primer: CAGTAACTGAGAGTTTACCCTTTGGT; reverse primer: CACACTAATGCTCTGGGAAGAAAGA; Cordova, TN). Results are reported as the relative difference between the cycle threshold values (CT) found in assays for the housekeeping and target gene of interest, raised to the second power to account for the doubling of target DNA during each cycle of the PCR process (i.e. Signal = 2 ^(CTcJUN – CTmSOD)^). ΔCT is an indication of transgene presence relative to the house keeping gene of choice.

### Collaborator derived G37R (29) progeny

During 2009, a colony of mice heterozygous for the G37R (line 29) locus were obtained from the laboratory of Dr. Neil Cashman where the transgene was maintained with a regular breeding cycle. Male animals positive for the transgene were maintained on a C57BL/6 background and crossed with naïve C57BL/6 dams to generate the requisite progeny. Employing multiple breeding pairs was done to limit the relatively rare event of meiotic recombination which could lead to a loss of tandem transgene hSOD1 copies. Once generated, the progeny were housed independently.

### Commercially derived G37R (29) progeny

In 2011, a total of six male B6.Cg-Tg(SOD1*G37R)29Dpr/J (Jackson Laboratory) breeders were crossed with 20 wild-type congenic C57BL/6 females (Jackson Laboratory) to generate progeny that seeded the colony. Females were utilized for 3 rounds of breeding to achieve adequate numbers of transgenic progeny. Due to facility space limitations, offspring were housed in groups segregated by sex. Various behavioural measures were assessed bi-weekly. For the purpose of this report, disease onset in this cohort was defined as the age at which subjects reached their peak body mass preceding the progressive weight loss that typifies ALS presentation in this model. This measure provided the earliest and most consistent measure of differentiation between transgenic and wild-type littermates. Additional parameters included recording the latency to fall from an elevated grid and assessing hind limb splay when the animal is suspended by the tail.

Both sets of progeny were originally a part of two separate studies assessing the therapeutic applicability of up regulating a neuronal growth factor in attenuating ALS pathogenesis (to be reported in a future correspondence). Briefly, at approximately 11 months of age, the collaborator derived progeny received bilateral delivery of a lentiviral vector carrying either a GFP (control group) or Progranulin (experimental group) construct into both gastrocnemius muscles. As a follow-up study, commercially derived progeny were exposed to a similar treatment paradigm at approximately 3.5 months of age. In either case, animals were monitored for behavioral outcomes and maintained until the humane endpoint was reached.

Comparisons between treatment (Progranulin) and control (GFP) groups did not establish a significant difference in terms of disease progression (Additional file 3: Figure S3). Consequently, for the purposes of this report, experimental and control animals from each progeny set were grouped to evaluate the influence of transgene dosage on lifespan and ALS presentation within the G37R(29) model.

### Statistical analysis

Data were analyzed utilizing the GraphPad Prism software platform (San Diego, CA). Differences in cycle threshold values between sourced animals were assessed with Kruskal-Wallis non-parametric analysis followed by Dunn’s Multiple Comparison test since not all assumptions for the parametric one-way ANOVA were upheld.

Differences in survival and onset of phenotype were assessed by the log rank test, with the requisite Bonferroni correction for multiple comparisons. All animals euthanized due to non-ALS pathologies such as severe dermatitis or abnormal growths etc., were excluded from survival analyses.

## Abbreviations

ALS: Amyotrophic lateral sclerosis; fALS: Familial ALS; sALS: Sporadic ALS; SOD1: Superoxide dismutase 1; hSOD1: Human superoxide dismutase 1, G93A, Glycine at codon 93 of SOD1 changed to alanine; G37R: Glycine at codon 37 of SOD1 changed to arginine.

## Competing interests

The authors declare that they have no competing interests that constitute a conflict of interest for both the content and interpretations contained within this report with the following exception: CAS is a co-founder of Neurodyn Inc.

## Authors’ contributions

PZ generated the colony of transgenic animals from the commercial breeders, maintained the colony, collected experimental data from behavioral assays, managed the statistical analysis, and wrote the first draft of the manuscript. GL generated the colony of collaborator derived animals, designed the study, wrote the protocol, collected experimental data from behavioral assays, and provided critical revisions integral to the manuscript. CAS conceptualized the study and participated in writing and revising the manuscript. All authors contributed to and have approved the final version.

## Supplementary Material

Additional file 1: Figure S1 Cycle threshold values of original commercial male breeders utilized in seeding our transgenic G37R (line 29) colony as assayed by Jackson laboratory. Results are plotted against CT values of historical control samples from G37R (line 29 and 42), G93A, and G85R animals. G37R (line 29) animals exhibit some of the lowest copy numbers of the hSOD1 transgene. Breeder males that seeded our colony were found to be well within a 0.5 deviation in CT values, and as such, are deemed to retain comparable levels of the transgene as historical G37R (line 29) controls (outlined region). Click here for file

Additional file 2: Figure S2 Proposed model explaining pre-clinical outcomes assessed in transgenic animals. It is possible the therapeutic effect (or lack thereof) seen in these animal models may primarily be a function of variations in mutant locus copy number and the corresponding diseased phenotype. Outliers at either end of the transgene expression spectrum will undermine replication studies and effective clinical translation. Click here for file

Additional file 3: Figure S3 Lack of treatment effect. Kaplan-Meier survival analysis indicated no treatment effect on the overall lifespan of transgenic G37R(29) animals in either cohort. Commercial male control vs. treatment p = 0.9666; Commercial female control vs. treatment p = 0.9120; Collaborator male control vs. treatment p = 0.5616; Collaborator female control vs. treatment p = 0.8613. Click here for file
