# Supplementary figures and images for: Reduction in hSOD1 copy number significantly impacts ALS phenotype presentation in G37R (line 29) mice: implications for the assessment of putative therapeutic agents
Source: J Negat Results Biomed. 2014 Aug 8;13:14. doi: 10.1186/1477-5751-13-14 (PMC4134475; doi:10.1186/1477-5751-13-14)

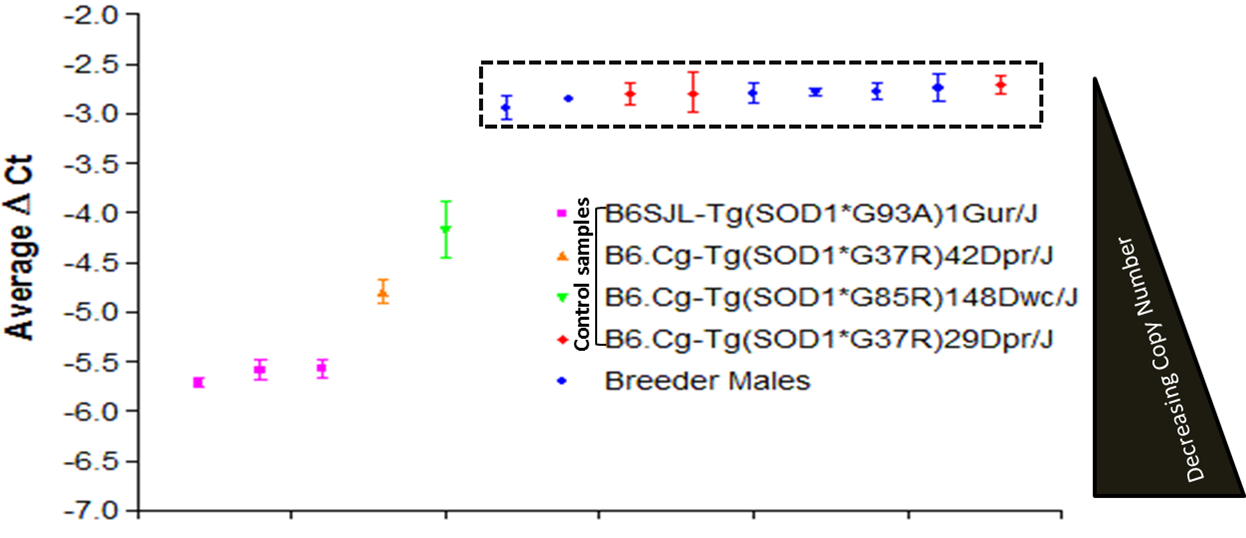

Supplement: Additional file 1: Figure S1 — Cycle threshold values of original commercial male breeders utilized in seeding our transgenic G37R (line 29) colony as assayed by Jackson laboratory. Results are plotted against CT values of historical control samples from G37R (line 29 and 42), G93A, and G85R animals. G37R (line 29) animals exhibit some of the lowest copy numbers of the hSOD1 transgene. Breeder males that seeded our colony were found to be well within a 0.5 deviation in CT values, and as such, are deemed to retain comparable levels of the transgene as historical G37R (line 29) controls (outlined region). [file 1477-5751-13-14-S1.png]

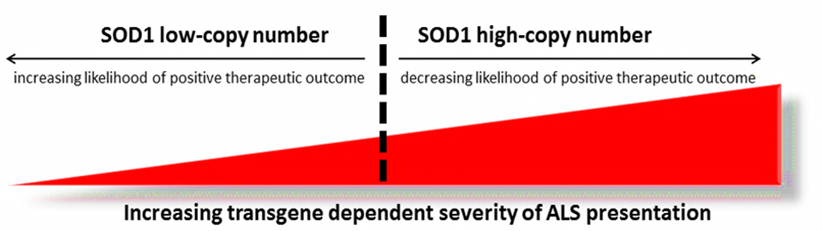

Supplement: Additional file 2: Figure S2 — Proposed model explaining pre-clinical outcomes assessed in transgenic animals. It is possible the therapeutic effect (or lack thereof) seen in these animal models may primarily be a function of variations in mutant locus copy number and the corresponding diseased phenotype. Outliers at either end of the transgene expression spectrum will undermine replication studies and effective clinical translation. [file 1477-5751-13-14-S2.png]

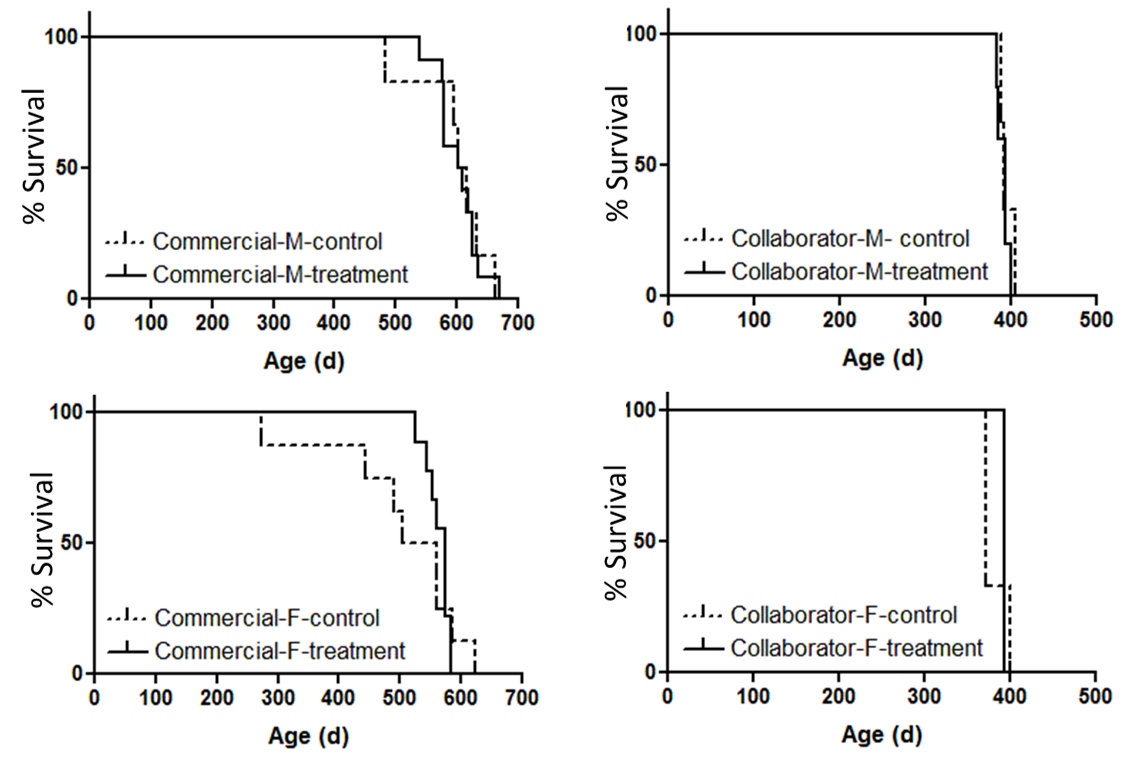

Supplement: Additional file 3: Figure S3 — Lack of treatment effect. Kaplan-Meier survival analysis indicated no treatment effect on the overall lifespan of transgenic G37R(29) animals in either cohort. Commercial male control vs. treatment p = 0.9666; Commercial female control vs. treatment p = 0.9120; Collaborator male control vs. treatment p = 0.5616; Collaborator female control vs. treatment p = 0.8613. [file 1477-5751-13-14-S3.png]
